# Anoikis-Related Genes Can Accurately Predict the Occurrence of Endometriosis: A Retrospective Cohort Study via Machine Learning Analysis

**DOI:** 10.1007/s10528-025-11151-x

**Published:** 2025-06-04

**Authors:** Lin Hong, Lan Zheng, Yu-Feng He, Ya-Xing Fang, Hui Chen, Kang-Jia Chen, Shu-Guang Zhou

**Affiliations:** 1 Department of Gynecology, Anhui Women and Children’s Medical Center Hefei, Maternal and Child Health Center of Anhui Medical University, The Fifth Affiliated Clinical College of Anhui Medical University, Hefei, 230001 Anhui China; 2 Department of Gynecology, Linquan Maternity and Child Healthcare Hospital, Fuyang, 236400 Anhui China

**Keywords:** Endometriosis, Anoikis, Nomogram, Diagnostic model, Machine learning

## Abstract

Endometriosis is one of the most common benign gynecological disorders, characterized by persistent pain and challenges with fertility. Anoikis, a specific form of apoptosis, occurs when cells detach from the extracellular matrix. Recent researches have revealed that anoikis resistance is the most important prerequisite for endometriosis development, but it's still not entirely apparent, yet, what part anoikis plays in endometriosis pathogenesis. We obtained the GSE141549 dataset from the GEO database, which served as our training cohort. Anoikis-related genes (ANRGs), sourced from the GeneCards database, were integrated with differentially expressed genes (DEGs) identified in GSE141549. Consequently, we obtained a collection of differentially expressed anoikis-related genes (DE-ANRGs). Subsequently, we employed machine learning algorithms to screen for key diagnostic DE-ANRGs and developed a nomogram model to predict and diagnose endometriosis. Ultimately, we validated our findings through in vitro experiments and an online endometriosis database. Within the training cohort, a total of 47 DE-ANRGs were identified. Furthermore, three machine learning methods pinpointed four diagnostic genes (CAV1, PDK4, CSPG4, SERPINE1). Based on these genes, we constructed a nomogram to facilitate the prediction and clinical diagnosis of endometriosis. To assess model's predictive accuracy, clinical adaptability, and discriminative ability. We performed calibration curves, Receiver Operating Characteristic curves and decision curve analysis. All assessments demonstrated our model's outstanding performance. Ultimately, consistent expression trends of four genes were observed in GSE7305 test cohort, clinical specimens, and the Turku database when compared to the training cohort. In addition, we also have revealed the immune landscape differences, which may offer new promising immunotherapeutic targets for endometriosis patients in the future. In addition to offering novel perspectives on the role of anoikis in endometriosis pathogenesis, our analysis also identified a panel of distinctive biomarkers with significant diagnostic potential.

**Graphical abstract:**

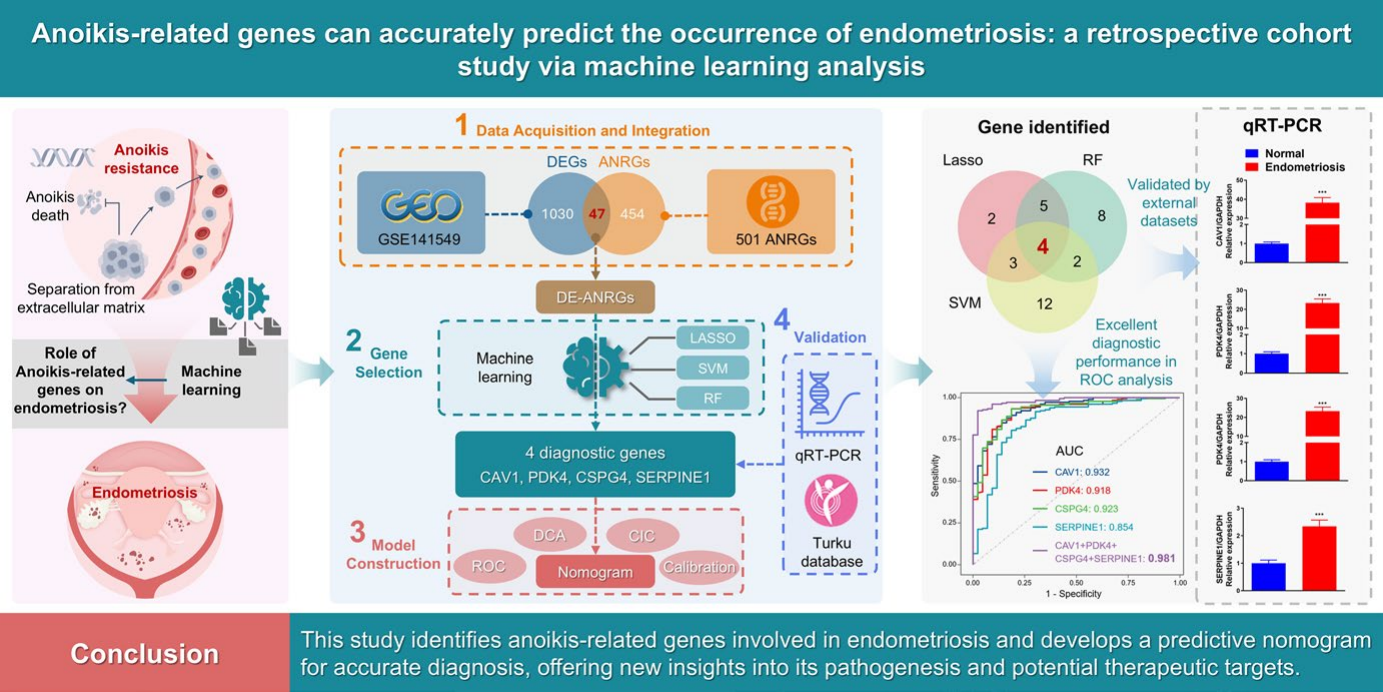

**Supplementary Information:**

The online version contains supplementary material available at 10.1007/s10528-025-11151-x.

## Introduction

Endometriosis (EM), one of the most prevalent disorders affecting females, is characterized by the aberrant proliferation of endometrial tissues outside the uterus, primarily involving ovaries and pelvic cavity (Bulun et al. 2019). Clinical characteristics of EM patients include dysmenorrhea, infertility, and persistent pelvic discomfort, all of which may have a significant detrimental effect on both physical and mental health during the childbearing years (Missmer et al. 2021). Epidemiological studies indicate that EM affects approximately 10–15% of women of reproductive age worldwide (Burney and Giudice 2012). Beyond its clinical manifestations, EM imposes substantial socioeconomic burdens, markedly reducing quality of life and potentially causing financial strain (Soliman et al. 2016). Nowadays, laparoscopic examination with histological confirmation remains the golden criterion for EM diagnosis. Nevertheless, surgical treatment entails risks, including diminished fertility, trauma, and adhesions (American College of Obstetricians and Gynecologists and American College of Obstetricians and Gynecologists 2018, Goncalves et al. 2021). The diagnostic delay of 8–12 years attributed to nonspecific symptomatology, elusive etiology, and the lack of reliable non-invasive biomarkers frequently results in missed therapeutic windows and suboptimal patient outcomes (Kiesel and Sourouni 2019, Rolla 2019).

Currently, the exact etiology and pathogenesis of EM patients is still incompletely understood (Laganà et al. 2019; Wang et al. 2020). The most widely accepted hypothesis, first proposed by Sampson in (1927), is the "menstrual blood reverse flow" theory (Burney and Giudice 2012), which was appealing and backed by several lines of scientific proofs(Sampson and Gynecology). This idea stated that shed endometrial cells can travel from the bilateral fallopian tubes into the abdominopelvic cavities in menstrual period. Notably, approximately 90% of healthy females undergoing laparoscopy during the peri-menstrual period were found to have menstrual blood present in their abdominopelvic cavities, supporting the prevalence of this phenomenon (Halme et al. 1984). Additional evidence for this aetiology comes from researches on outflow tracts obstruction, which indicated that teenage girls with outflow obstruction are more likely to develop EM (Sanfilippo et al. 1986).

Apoptosis, a critical mechanism for eliminating regurgitated endometrial cells in the peritoneal cavity, has been shown to influence endometriosis progression (Kobayashi et al. 2024). A type of programmed apoptosis known as anoikis, which occurs when cells detach from the extracellular matrix (ECM) (Adeshakin et al. 2021; Wang et al. 2022). Anoikis serves to efficiently eradicate misplaced cells during normal development, thereby preventing abnormal attachments. However, the deregulation of anoikis-related genes (ANRGs) can influence diverse ailments. For instance, resistance to anoikis facilitates the metastasis of tumors in the pancreas, liver, and lungs (Taddei et al. 2012; Guizhen et al. 2022; Kang et al. 2022; Bose et al. 2023). Furthermore, anoikis has also been related to nontumor disorders such as inflammatory bowel disease, cardiovascular illness, and neurological disorders (Gary and Mattson 2001, Michel 2003, Zhang et al. 2023). Recent studies indicate that the endometrium of patients with EM displays decreased levels of pro-apoptotic factors while elevated levels of anti-apoptotic factors compared to healthy female's endometrial tissues (Taniguchi et al. 2011). Consequently, we hypothesize that endometriosis pathogenesis may involve acquired resistance to anoikis.

In the present work, our objective was to explore any possible relationship between the onset of EM and Anoikis, as well as the contributions of DE-ANRGs to the prospective immunotherapeutic targets and diagnostic tools.

## Materials and Methods

### Data Acquisition and Preparation

**Fig. 1:**
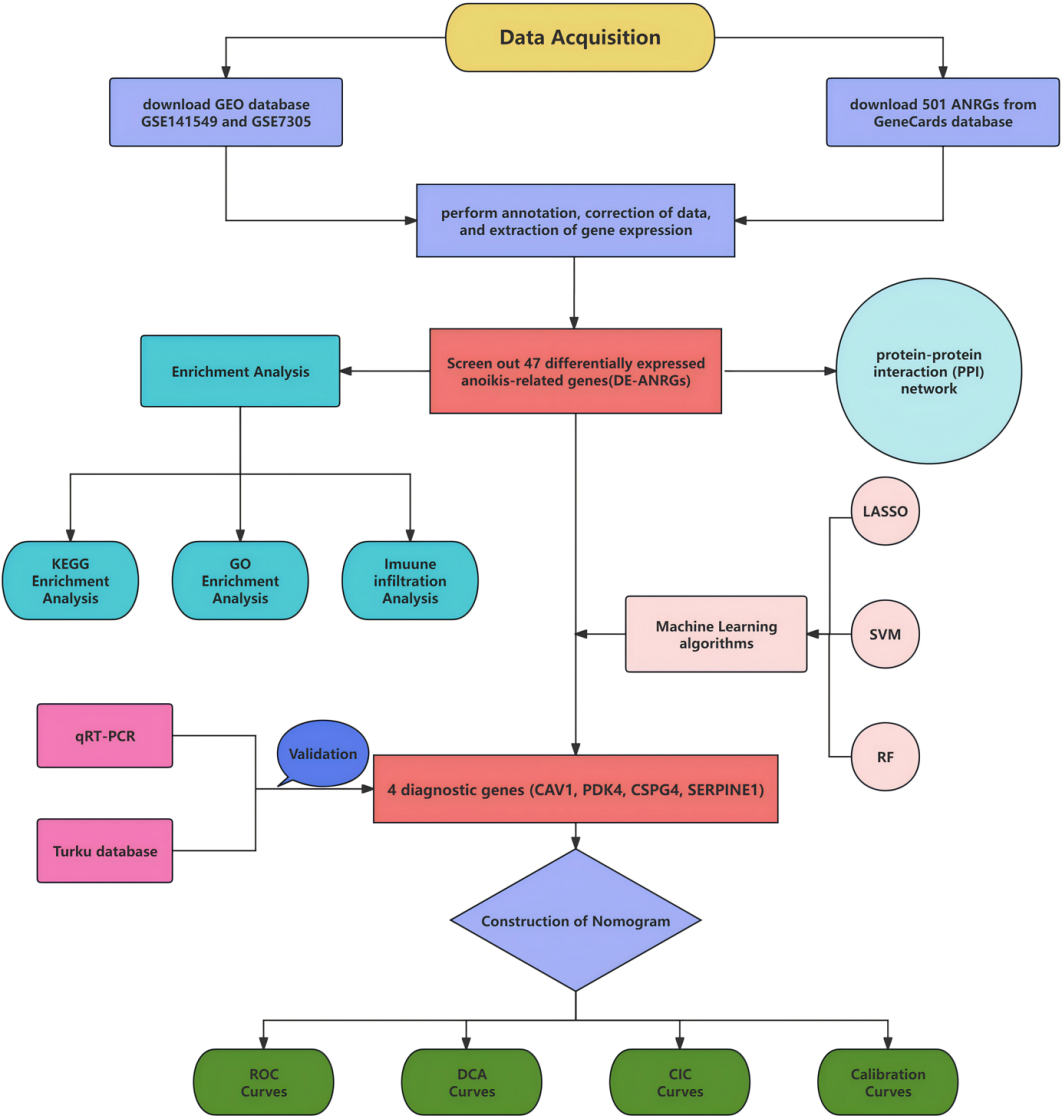
A corresponding design flow-chart consistent with our study

We downloaded GSE141549 and GSE7305 Series Matrix data files from the GEO database (http://www.ncbi.nlm.nih.gov/geo). The GSE141549 profiles yielded 179 endometriosis cases and 43 control samples, while GSE7305 comprised 10 endometriosis specimens and 10 control specimens. For the training and validation sets, GSE141549 and GSE7305 were employed, respectively. Additionally, anoikis-related genes were acquired via the online GeneCards database (https://www.genecards.org/). 501 ANRGs were selected based on |correlation coefficient|> 0.4 after being extracted from the database. The study design flowchart for our research is presented in Fig. 1.

### DE-ANRGs Identification

The "limma" program was employed to obtain the differentially expressed genes (DEGs) in GSE141549 dataset. These genes met the cutoff criterion of adj *p* value < 0.05 and |log2 (fold change) |> 1. The intersection of DEGs and ANRGs was then identified using a Venn diagram.

### Analysis of Functional Enrichment

The DE-ANRGs were evaluated employing KEGG analysis of biological pathways and GO enrichment analysis. The annotation and visualization were performed by the "clusterProfiler" and "GOplot" R packages.

### Construction and Visualization of Protein–Protein Interaction Network

Applying the STRING online database (https://cn.string-db.org/), the DE-ANRGs' PPI network was created. Following that, we visualized the results via Cytoscape software (version 3.10.1).

### Screening Diagnostic Genes by Machine Learning Approaches

The diagnostic DE-ANRGs in our research were identified using three machine learning approaches. To ascertain the best possible penalty value with the least binomial deviation, we used "glmnet" R tool for LASSO regression analysis. Employing such as "caret","e1071", and “kernlab" R packages, the SVM-RFE method was applied, and an investigation was conducted into the result with the least cross-validation error. In addition, R package "randomForest" was used to find the least amount of error in a level.

### Establishing and Verifying the Nomogram

Multivariate logistic regression was utilized to create the nomogram; with the regression coefficient indicating the contribution of each impacting element to the model's dependent variable. Numerous predictors are integrated, and each influencing factor is given a score. The graph is then plotted using a line segment with a scale in accordance with a specific proportion.

Calibration curves, Receiver Operating Characteristic curves and decision curve analysis were employed to further assess our model's diagnostic efficacy.

### Evaluation in Immune Landscape of Endometriosis

By applying the CIBERSORT method, the proportion of twenty-two immune cells were estimated. Relative percentages of these immune cells for each tissue were assessed. The "corheatmap" package was utilized to generate visual representations that depict the correlation between four diagnostic DE-ANRGs' relative expression level and different immune cells' infiltration abundance.

### Collection of Human Tissues

We recruited a total of 10 patients from the Department of Gynecology, Linquan Maternity and Child Healthcare Hospital between September 2024 and October 2024. Five samples in the experimental group were endometriosis tissues obtained from chocolate cysts. The control group consisted of five tissues from the endometrium of clinical specimen who treated surgically for fibroids in the uterus.

### qRT-PCR for Diagnostic Genes Validation

Following their freezing in liquid nitrogen, total RNA was extracted from five endometriosis and five normal specimens applying the TRIzol® reagent (15,596,026, America). Reverse transcription was carried out at 42 °C for 15 min, followed by 3 min at 95 °C. The Talent qPCR PreMix (FP209-02, China) was then employed in a 10 ml SYBR reaction mixture. The protocol included 40 cycles at 95 °C for 5 s and at 60 °C for 15 s, coupled with an initial cycle at 95 °C lasting 3 min. Then, the wanted sequences of mRNA with the best melting curves and sizes were found. Table 1 shows four primer sequences that were employed in PCR process. Primer specificity was confirmed by melt curve analysis, and primer sequences were selected based on previously validated studies.

**Table 1:** Four primer sequences that were employed in PCR process

DE-ANRGs	Sequences
CAV1	F:5'-GCTTCACCACCTTCACTGTG-3' R:5'- GCAGGAAAGAGAGAATGGCG-3'
PDK4	F:5'-CAGACAGAGGAGGTGGTGTT-3' R:5'-CCGTAACCAAAACCAGCCAA-3'
CSPG4	F:5'-GAGGGCAAGGAGAAGGAAGT-3' R:5'-CTGGGTTGGAGTGGAAAAGC-3'
SERPINE1	F:5'-AAGCCTAATCAGCCCACCAT-3' R:5'-CACCGTCCAGTGCAAAATCA-3'

### Turku Database-Based Validation

An online endometriosis database called Turku combines clinical data and gene expression profiles from 115 EM patients and 53 normal control samples. It was utilized to verify our preliminary findings.

### Statistical Analysis

R software was applied in our research to analyze all data (version 4.3.1). Student's *t*-test was applied to analyze continuous variables. If *p* value was < 0.05, then it was considered to have statistical significance for above analyses.

## Results

### Screening for DE-ANRGs

**Fig. 2:**
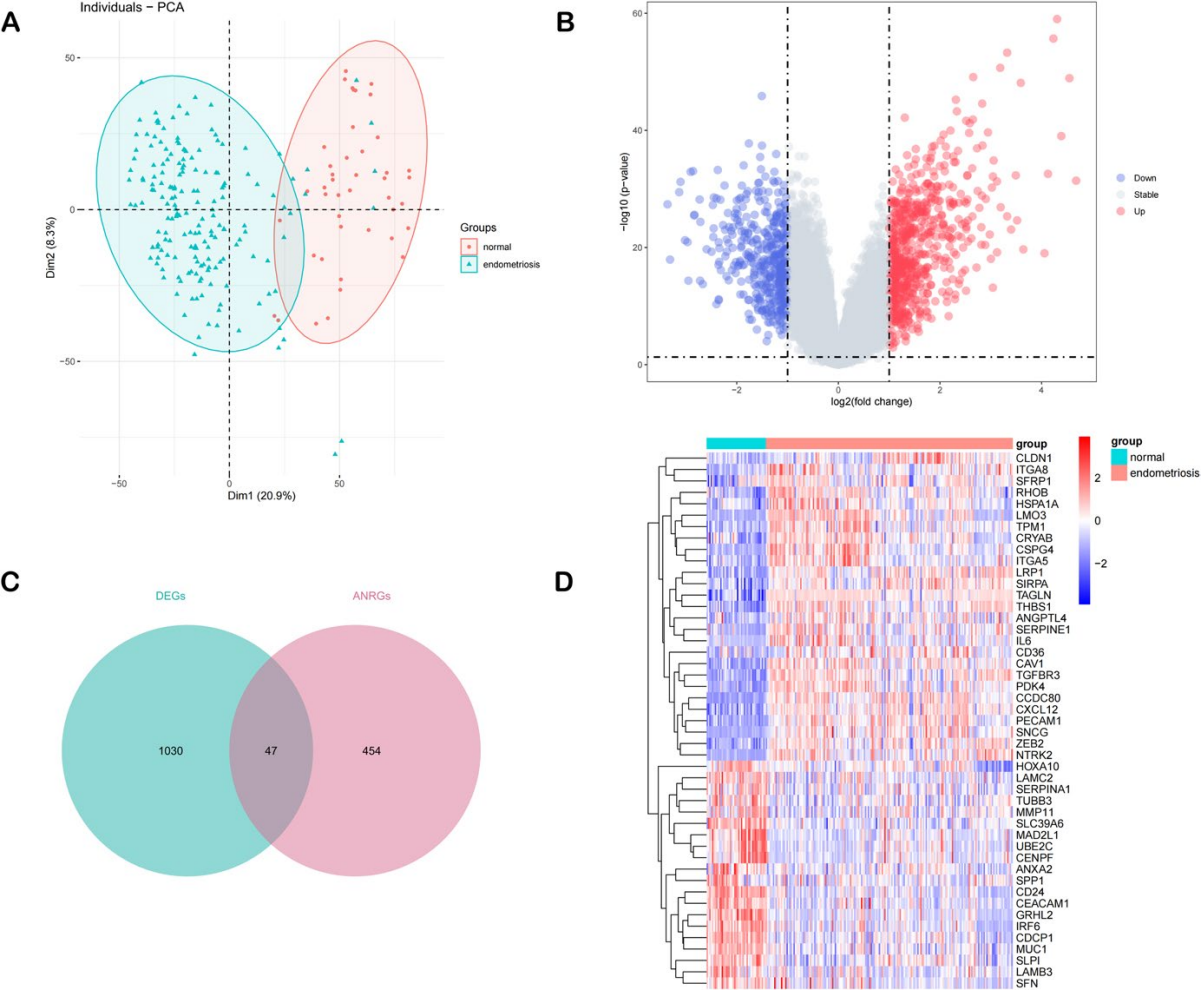
Screening and identification of DE-ANRGs. Principal component analysis: red denotes the Normal dataset, and blue denotes the Endometriosis dataset (**A**). Volcano plot: the color gray signifies genes that are not expressed differently, while the colors red and blue denote differentially expressed genes that are upregulated and downregulated, respectively (**B**). Venn diagram: 47 DE-ANRGs are shown (**C**). Heatmap of DE-ANRGs (**D**) (Color figure online)

We used the GSE141549 dataset to conduct a retrospective analysis of 179 endometriosis cases and 43 control samples. Initially, we performed Principal Component Analysis (Fig. 2A) to explore potential differences between the endometriosis and control samples. Subsequently, it revealed a total of 1077 DEGs filtered from the GSE141549 cohort (Fig. 2B). By intersecting these DEGs with 501 ANRGs, we identified 47 DE-ANRGs (Fig. 2C). Comparative analysis revealed distinct expression patterns of these 47 DE-ANRGs between groups. And 27 DE-ANRGs (including CAV1, PDK4, CSPG4, and SERPINE1) were found to be upregulated in the EM patients, while the remaining 20 DE-ANRGs exhibited upregulation in the normal individuals (Fig. 2D).

### Analysis of Functional Enrichment

**Fig. 3:**
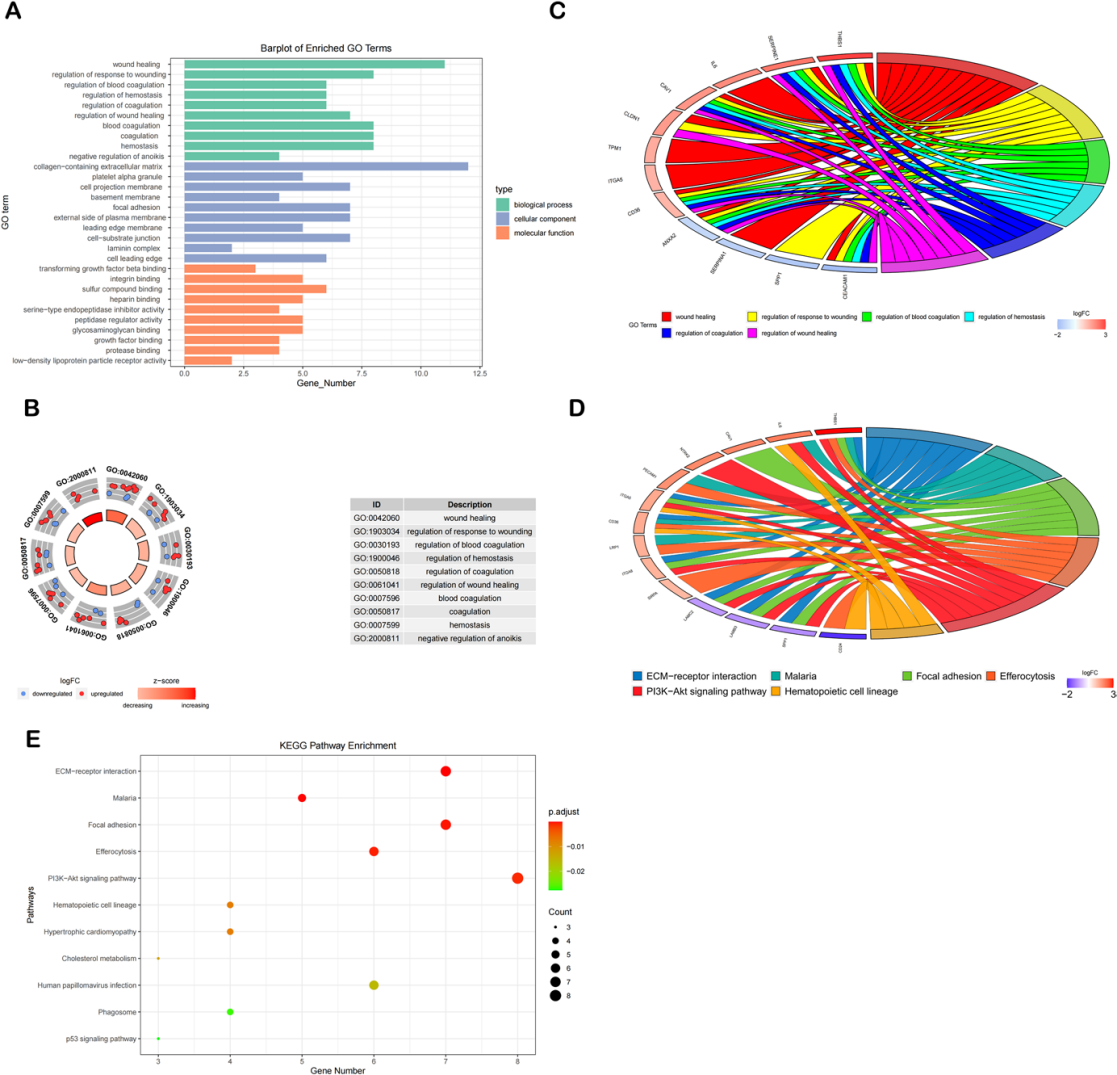
Performing Gene Ontology and KEGG enrichment analysis on DE-ANRGs

GO & KEGG enrichment analyses were executed to elucidate the organismal characteristics of DE-ANRGs. As for GO functional enrichment results, the DE-ANRGs were considerably abundant in, according to BP analysis, negative regulation of anoikis, wound healing, blood coagulation, hemostasis, coagulation (Fig. 3B). In CC, collagen-containing extracellular matrix, cell projection membrane, focal adhesion were the pathways with the highest level of enrichment. Sulfur compound binding, glycosaminoglycan binding and integrin binding were notable in the MF analysis (Fig. 3A). Additionally, DE-ANRGs were involved in various KEGG pathways, including focal adhesion, interactions between the extracellular matrix and receptors, and the PI3K-Akt signaling pathway (Fig. 3D, E). Furthermore, the genes corresponding to the six principal biological process items are illustrated as Fig. 3C.

GO findings of the DE-ANRGs study to display the MF, CC, and BP enrichment results (**A**). Displaying the top 10 DE-ANRGs in BP using a circle diagram (**B**). KEGG enrichment analysis results (**C**, **D**). The top 6 BP terms and their corresponding genes (**E**)

### PPI Network for DE-ANRGs

**Fig. 4:**
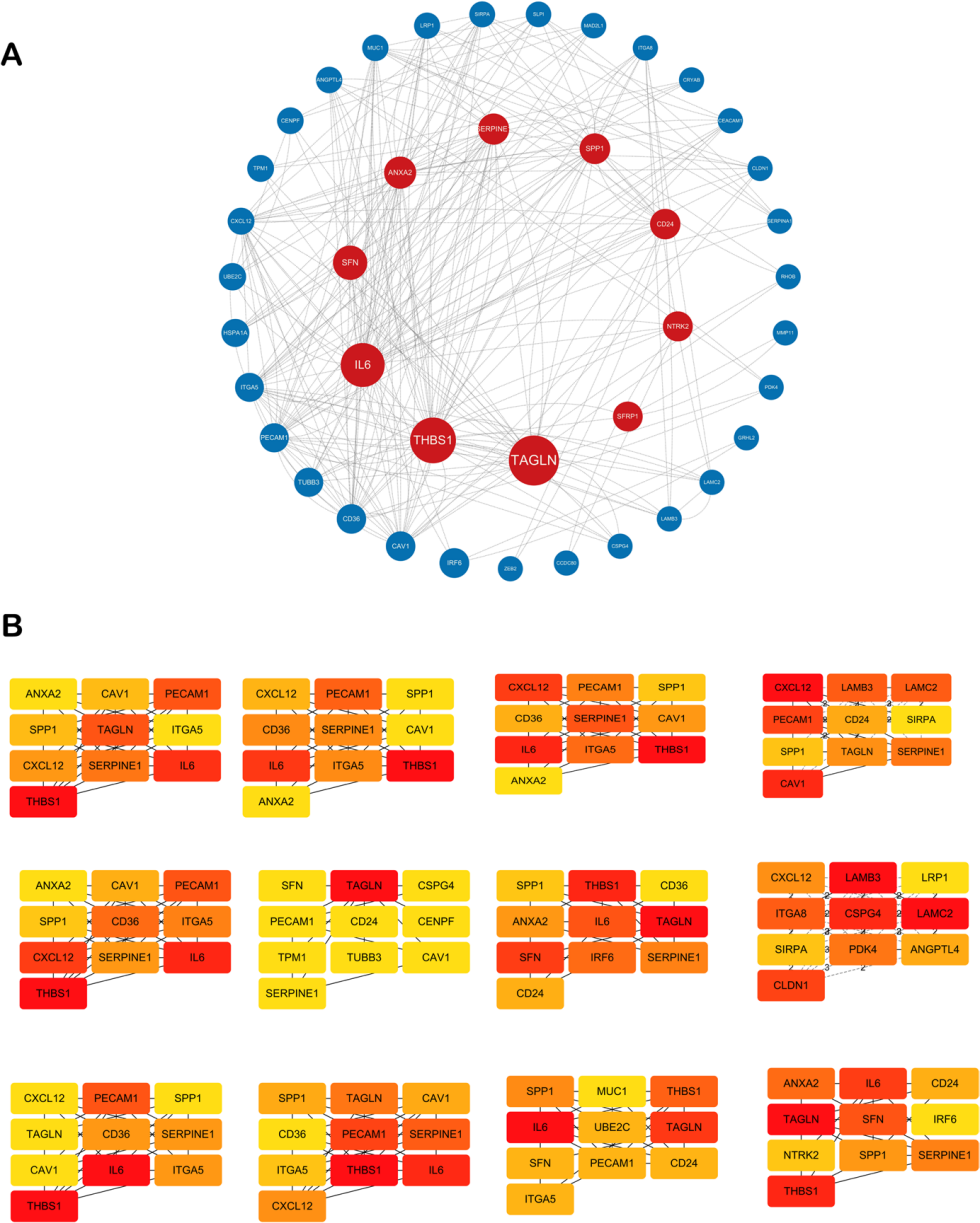
PPI network for DE-ANRGs. The PPI network of 47 DE-ANRGs was visualized using Cytoscape (**A**). Top ten genes screened using 12 algorithms by Cytohubba plug-in **B**

Our PPI network was constructed by a total of 47 DE-ANRGs and visualized in Fig. 4A. Furthermore, we employed the Cytohubba tool to identify the 10 most critical genes within the network by employing 12 distinct methods (Fig. 4B).

### Identifying Diagnostic Genes by Machine Learning Approaches

**Fig. 5:**
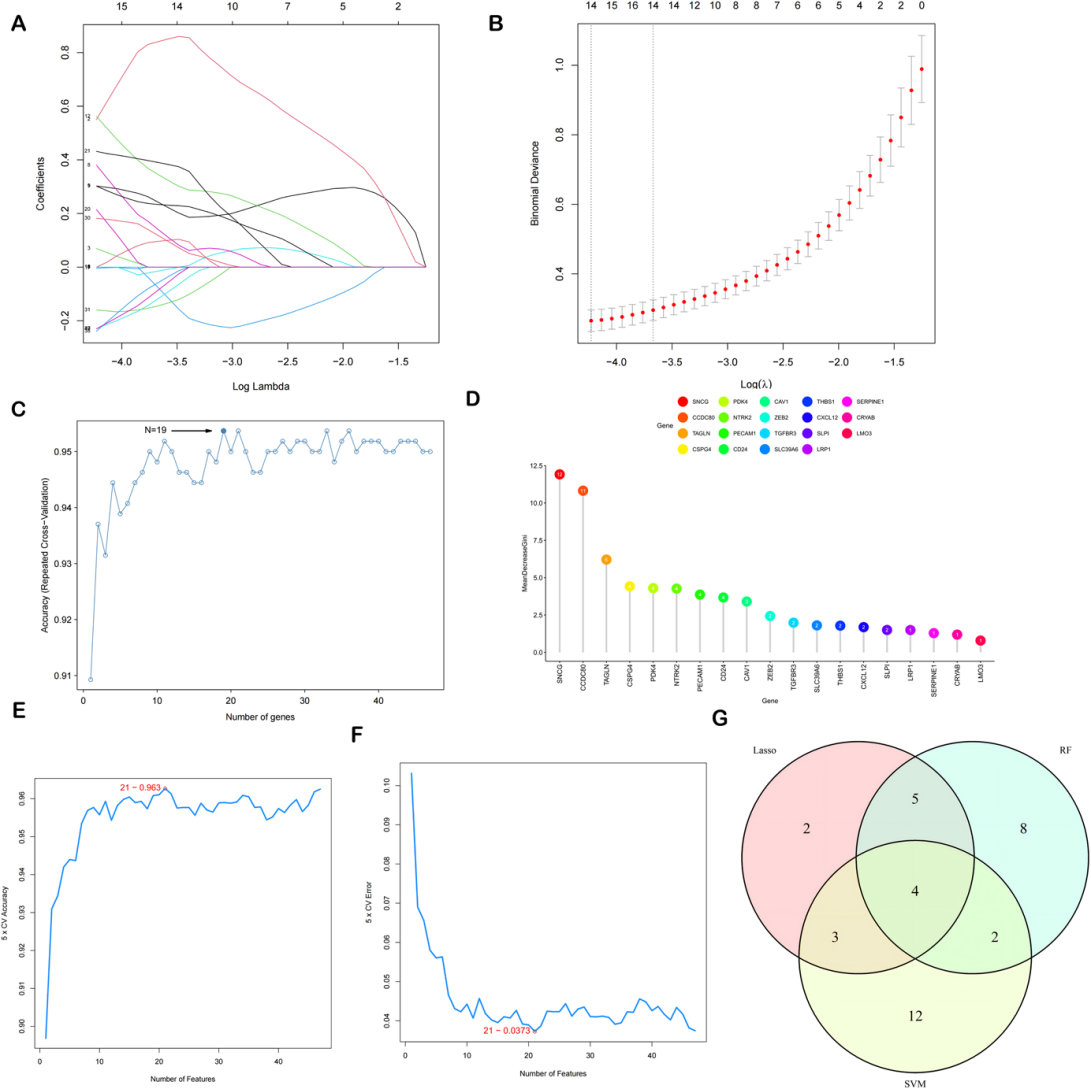
Four key DE-ANRGs were chosen as diagnostic biomarkers for EM by machine learning algorithms. DE-ANRGs' LASSO coefficients and the dotted lines in the LASSO regression represent the optimum log (*λ*) value (**A**, **B**). The mean decrease gini was used to rank the 19 genes that the RF found to have the best accuracy (**C**, **D**)

We utilized three different machine learning approaches: LASSO, RF, and SVM-RFE. For the LASSO regression algorithm, it revealed 14 potential diagnostic genes (Fig. 5A, B). The Mean Decrease Gini was used by the RF to rank alternative genes, and the 19 genes with the best accuracy were selected (Fig. 5C, D). SVM-RFE identified 21 diagnostic genes (Fig. 5E, F). Ultimately, the outcomes were intersected by using a Venn diagram (Fig. 5G).

### Building and Verifying the Diagnostic Nomogram

**Fig. 6:**
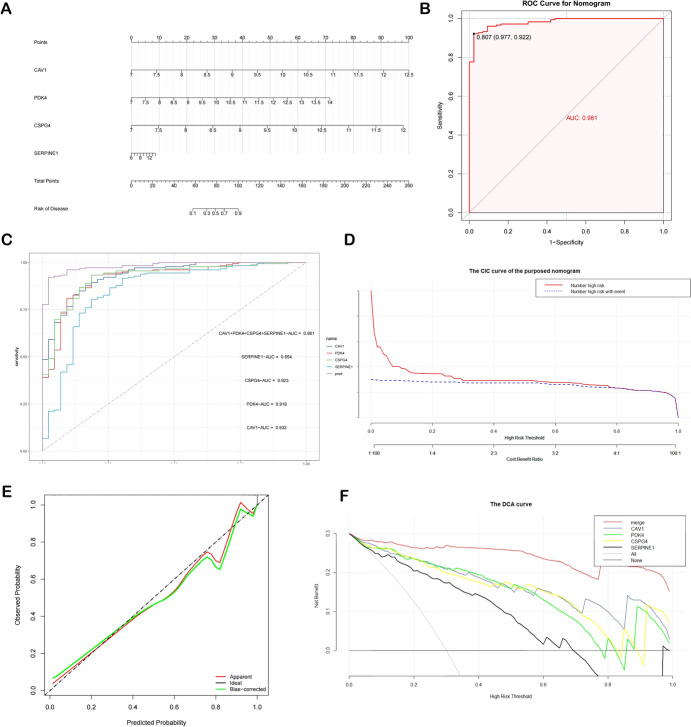
Building and verifying the nomogram. A nomogram was developed using the expression of four chosen diagnostic genes (**A**). ROC curves for the all-model genes and nomogram for EM patients (**B**, **C**). The clinical efficacy of the nomogram was evaluated using Calibration curves, DCA curves, and CIC curves (**D–F**)

To facilitate the four diagnostic genes' clinical applicability in our study, we then established a nomogram (Fig. 6A). ROC curves were also created to assess our nomogram's predictive accuracy (Fig. 6B). The AUCs of following diagnostic genes in the GSE141549 are listed as: CAV1: AUC = 0.932, PDK4: AUC = 0.918, CSPG4: AUC = 0.923, SERPINE1: AUC = 0.854. Notably, the AUC for nomogram model was the highest (AUC = 0.981), demonstrating that the nomogram provided more precise and accurate predictions (Fig. 6C). In addition, we employed the clinical impact curve (CIC) as well as Calibration curve and decision curve analysis, which indicated that employing the EM diagnostic nomogram model had a higher net benefit and precision (Fig. 6D–F).

The SVM method selected 21 genes (**E**, **F**). LASSO, RF, and SVM algorithms' feature genes are displayed in a Venn diagram (**G**).

### Verification of Analysis Outcomes in GSE7305 Dataset

**Fig. 7:**
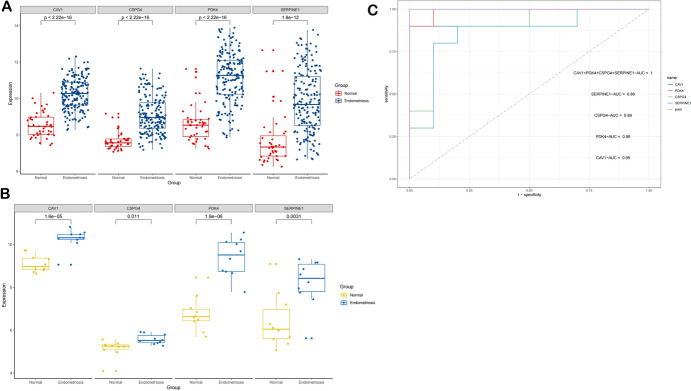
Assess and analyze the differential expression and diagnostic value of four key genes. Expression evaluation of CAV1, PDK4, CSPG4 and SERPINE1 in EM and Normal samples in GSE141549 (**A**). Expression evaluation of four diagnostic genes in GSE7305 (**B**). ROC curves for the all-model genes and nomogram for EM patients in test set GSE7305 (**C**)

The GSE141549 dataset revealed significant differential expression of four diagnostic genes. As illustrated in Fig. 7A, EM samples exhibited increased expression of CAV1 (*P* < 2.22e−16), CSPG4 (*P* < 2.22e−16), PDK4 (*P* < 2.2e−16), and SERPINE1 (*P* < 1.8e−12) compared to control samples. The diagnostic gene expression levels in the testing dataset GSE7305 were consistent with those observed in GSE141549, as demonstrated in Fig. 7B. ROC curve analysis in the test cohort demonstrated exceptional diagnostic performance for all four biomarkers (Fig. 7C): CAV1: AUC = 0.95, PDK4: AUC = 0.99, CSPG4: AUC = 0.89, SERPINE1: AUC = 0.86.

### Immune Landscape Evaluation for Endometriosis

**Fig. 8:**
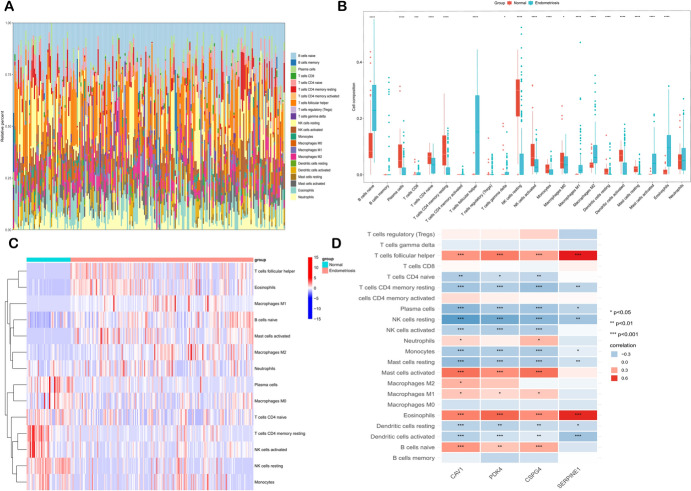
Correlation of diagnostic genes with immune cell infiltration. **A** stacked plot visualized the extent of immune cell infiltration in EM samples (**A**). Heatmap and boxplots showing comparison of immune cell infiltration in EM and normal samples (**B**, **C**). The heatmap visualized the correlation of four diagnostic genes with immune cells (**D**)

The pathogenesis of EM was thought to be primarily affected by an aberrant immune environment as well as an unbalanced distribution of immune cells. To investigate this further, we applied CIBERSORT approach to assess the proportion of 22 immune cells in EM tissues. The infiltration levels of specific immune cells in these samples were comprehensively illustrated using a stacked plot (Fig. 8A), where the total fraction of immune cells summed to 1.Box plots and a heatmap showed that patients with EM exhibited increased levels of M2 macrophages, CD8 T cells, naive B cells, M1 macrophages, activated mast cells, follicular helper T cells, and eosinophils, while showing decreased levels of monocytes, activated NK cells, resting memory CD4 T cells, resting NK cells, plasma cells, and naive CD4 T cells, (Fig. 8B, C). Furthermore, as depicted in Fig. 8D, we identified strong correlations between the four diagnostic genes and immune cell populations in EM patients.

### Verification of Diagnostic Genes via qRT-PCR and Turku Database

**Fig. 9:**
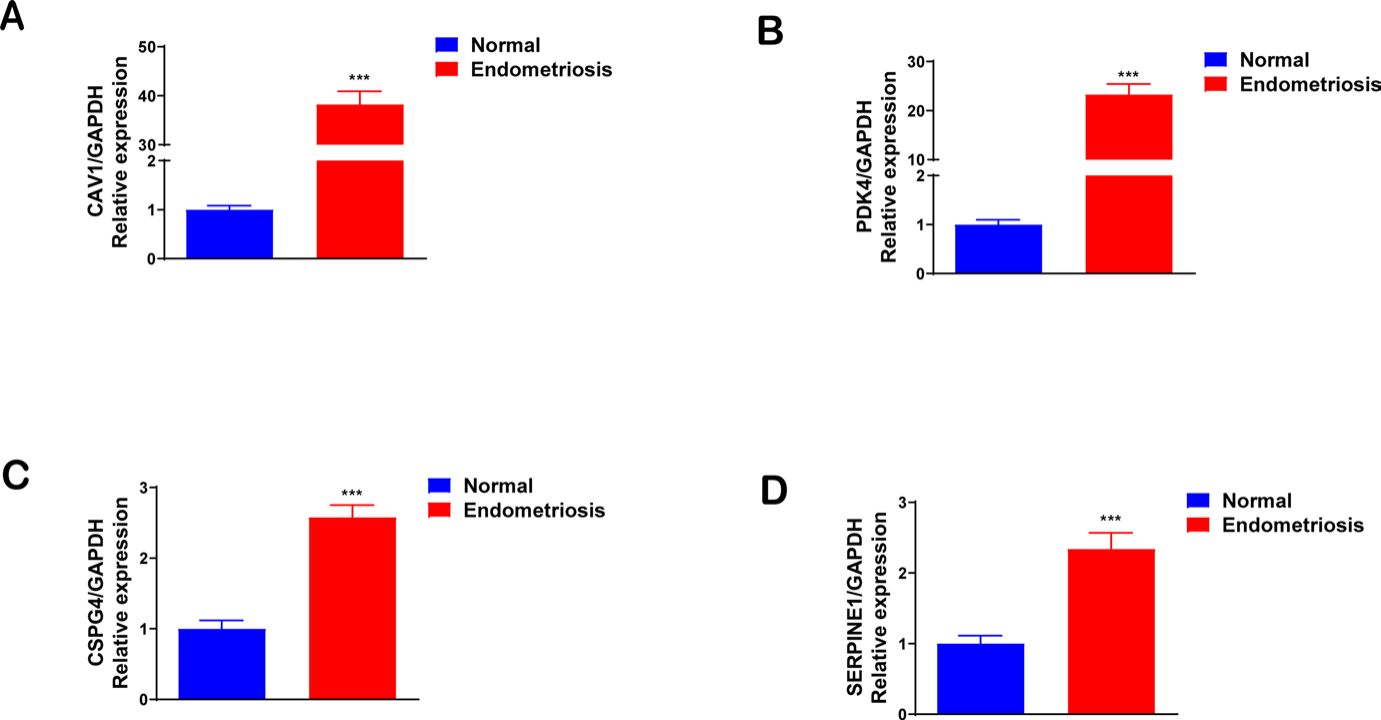
The expression level of four diagnostic biomarkers in normal endometrial tissues and endometriosis tissues

**Fig. 10:**
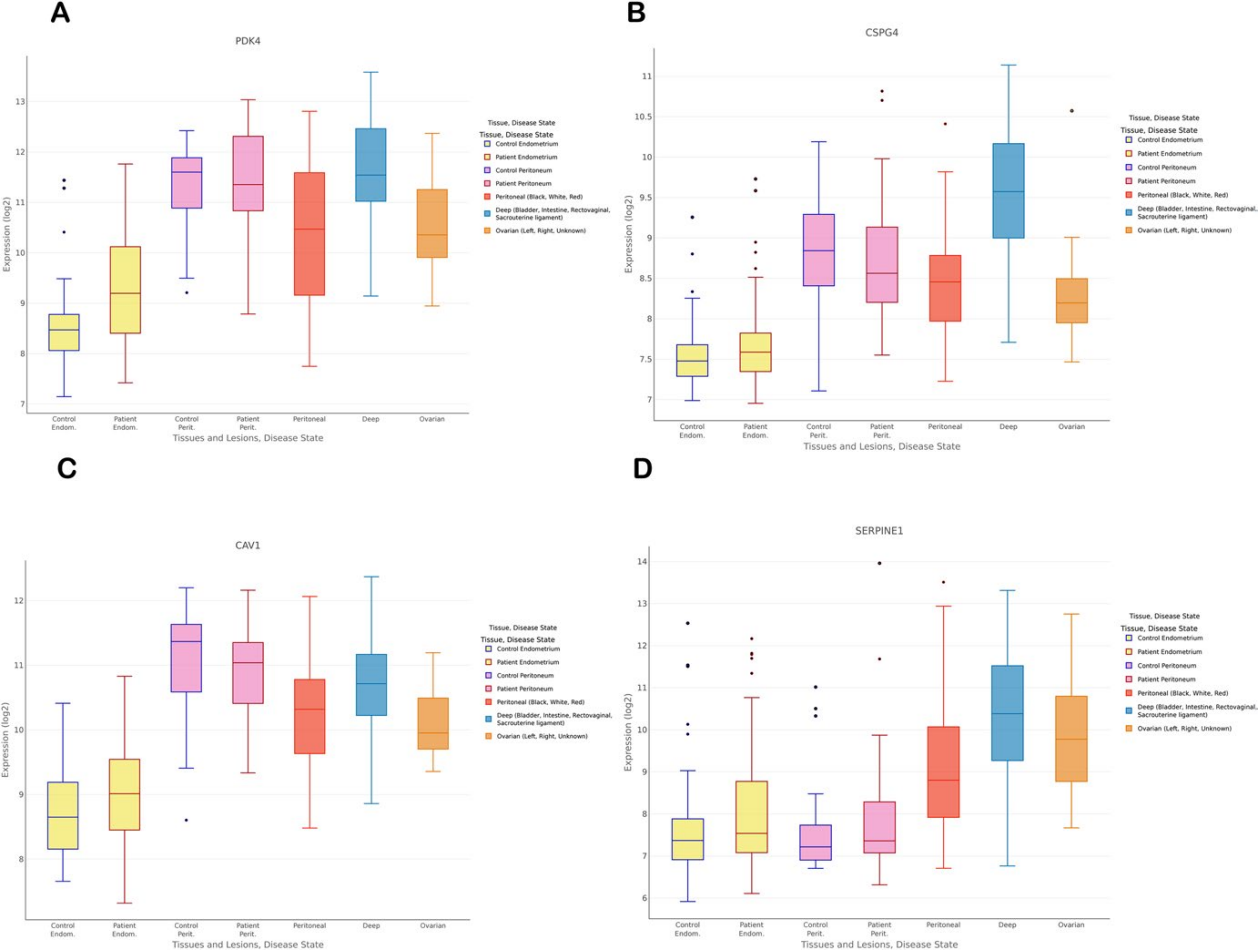
Assessing the validity of our studies via the Turku online endometriosis database. the expression level of four diagnostic biomarkers respectively (**A–D**)

To validate the four diagnostic genes ' expression in our nomogram model, we employed qRT-PCR in clinical samples. The results were illustrated in Fig. 9. Additionally, the accuracy of our findings was evaluated using the Turku database as well (Fig. 10).

## Discussion

EM is a prevalent gynecological disorder in reproductive age worldwide (Mortlock et al. 2022), with an estimated global burden exceeding 200 million cases (Rogers et al. 2009). Researches indicated that EM shares characteristic features with malignant neoplasms in proliferating, invading, and reoccurring (Tan et al. 2022; Guo et al. 2023). For the diagnosis of EM, surgery with postoperative pathological examination is regarded as the golden standard (Pei et al. 2023). Nevertheless, a major challenge in clinical practice is the delay in diagnosing EM, which not only raises the likelihood of missing optimal treatment opportunities but also accelerates the progression of EM stages (Saunders and Horne 2021; Horne and Missmer 2022; Jiang et al. 2022). Thus, investigating the EM' s fundamental mechanism and discovering reliable, non-invasive diagnostic biomarkers are critical clinical priorities.

Anoikis, which means "homelessness" in Greek, was deemed as a specific way that cell dies. It occurs when normal cells separated from the ECM (Frisch and Francis 1994). A hallmark characteristic of malignant progression is the acquisition of anoikis resistance (Chen et al. 2023). Researches showed that EM and malignant tumors share several biological characteristics, including proliferation, invasion, and recurrence. Besides, Endometrial tissues of EM patients exhibit reduced levels of pro-apoptotic factors and elevated levels of anti-apoptotic factors compared to the endometrial tissues of healthy females. Consequently, we believed that EM patients may have developed some anti-anoikis mechanisms.

We conducted an exploratory analysis of ANRGs to enhance our understanding of the pathophysiology of EM. Functional enrichment studies indicated that 47 DE-ANRGs are predominantly associated with processes such as wound healing, negative regulation of anoikis, extracellular matrix containing collagen, PI3K-Akt. Negative regulation of anoikis, a biological process that means resistance to anoikis, is crucial in the development and metastasis of many malignancies (Paoli et al. 2013). This finding suggests that anti-anoikis may have a role in the EM's development. Therefore, comprehending the molecular processes related with anoikis is crucial for developing effective treatments for EM patients (Wang et al. 2024). Furthermore, DE-ANRGs may influence wound healing, a process that involves invasiveness and migratory capabilities which are essential to EM. Collectively, these findings highlight anoikis as a potential contributor and therapeutic target in EM.

Recently, machine learning has been effectively implemented across various fields. In our study, we employed three machine learning approaches: SVM-RFE, LASSO and RF to screen for key DE-ANRGs. Ultimately, we identified four diagnostic genes (CAV1, PDK4, CSPG4, SERPINE1) as potential candidate biomarkers, which were incorporated into a nomogram for endometriosis diagnosis.

Nomograms are an approachable, graphical mathematical method that uses biological or clinical data to estimate the probability of clinical events for a specific person,, such as the onset or course of a disease (Iasonos et al. 2008). In this study, we showcased our model's remarkable predictive capability. Besides, the Calibration curves displayed a high level of consistency between predicted probabilities and the real occurrence rates. Decision Curve Analysis(DCA) was created by Vickers and Elkin (Vickers and Elkin 2006) to assess predictive models' clinical practicability through threshold probability assessment. Our established nomogram was further supported by its adoption of DCA.

Recent research has suggested that dysregulation of immune system is a key factor in the emergence and progression for EM (Yang et al. 2023).Anoikis enables cancerous cells to evade the immune system's defense in various tumors (Zhang et al. 2024). However, how anoikis affects the immune system during the development of EM remains unclear. Our findings demonstrated that noteworthy differences (*P* < 0.05) were noted in the infiltration abundance of 18 immune cells in EM and normal individuals. Our findings also indicated a potential relationship between the pathophysiology of EM and increased levels of activated mast cells, CD8 and follicular helper T cells, M1 and M2 macrophages, eosinophils, and naive B cells, alongside decreased infiltration of plasma cells, CD4 naive T cells, CD4 memory resting T cells, resting and activated NK cells, and monocytes.M2 macrophages were extensively studied for their crucial involvement in immunosuppression and neuroangiogenesis in the context of EM (Wu et al. 2017). Mast cells (MCs) are inherent constituents of female uterus, and their stimulation contributes to regulating proper menstruation by facilitating the shedding of the endometrium (Menzies et al. 2011). Endometriotic lesions exhibited a higher recruitment of mast cells compared to normal endometrium (Anaf et al. 2006). Additionally, a research using rat model demonstrated that MCs activation contributes to the enhancement of growth in endometriotic lesions (Lin et al. 2015). However, the potential therapeutic efficacy of MC inhibitors for endometriosis needs additional verification through standardized clinical trials (Binda et al. 2017). Natural Killer (NK) cells function as sentinels, providing protection against exogenous and deleterious entities. Current evidence suggests that Natural Killer (NK) cells may play a critical role in clearing retrograde menstrual endometrial debris, while impaired NK cell activity in the peritoneal cavity could facilitate the survival, implantation, and subsequent growth of ectopic endometrial cells (Du et al. 2017).

This study represents the first comprehensive investigation integrating Anoikis and Endometriosis through advanced machine learning approaches. Our developed nomogram not only provides a clinically applicable diagnostic tool but also enables risk stratification. Furthermore, our comprehensive analysis of functional pathways and immune microenvironment characteristics in EM patients revealed distinct molecular signatures and immune dysregulation patterns, highlighting promising targets for immunotherapeutic intervention. Nevertheless, our study has several limitations that warrant consideration. First, while our analysis leveraged publicly available genomic datasets, the sample size for clinical validation remains relatively limited. Future studies with expanded patient cohorts will be essential to enhance the robustness of our findings. Second, although enrichment analysis identified several molecular pathways potentially involved in endometriosis pathogenesis, the precise mechanistic roles of the identified DE-ANRGs in disease development remain unclear and require further functional validation. Third, while our preliminary immune infiltration analysis revealed distinct immune cell profiles between patients and controls, deeper investigation of immune-related pathways and their therapeutic implications is needed. Addressing these limitations through expanded clinical cohorts and mechanistic studies will be critical for translating these findings into clinical applications.

## Conclusion

In conclusion, this study identified four diagnostic biomarkers (CAV1, PDK4, CSPG4, and SERPINE1) and developed a machine learning-based nomogram with high predictive accuracy for endometriosis. Furthermore, our comprehensive analysis of the endometrial immune microenvironment has provided some insights into disease pathogenesis and revealed potential therapeutic targets. Collectively, our research provided novel perspectives on the development of EM and identified new possible targets for clinical treatment as well as diagnostic tools.

## Supplementary Information

Below is the link to the electronic supplementary material.

Supplementary file1 (DOCX 2121 KB)

## Data Availability

No datasets were generated or analysed during the current study.

## Abbreviations

EM: Endometriosis
GEO: Gene expression omnibus
DEGs: Differentially expressed genes
ANRGs: Anoikis-related genes
DE-ANRGs: Differentially expressed anoikis-related genes
GO: Gene ontology
KEGG: Kyoto encyclopedia of genes and genomes
LASSO: Least absolute shrinkage and selection operator
RF: Random forest
SVM-RFE: Support vector machine-recursive feature elimination
ROC: Receiver operating characteristic
qRT-PCR: Reverse transcription and quantitative real-time polymerase chain reaction
DCA: Decision curve analysis
CIC: Clinical impact curve
ECM: Extracellular matrix
BP: Biological process
MF: Molecular function
CC: Cellular component
PPI: Protein–protein interaction networks
AUC: Area under the curve
