# Supplementary material for: Anoikis-Related Genes Can Accurately Predict the Occurrence of Endometriosis: A Retrospective Cohort Study via Machine Learning Analysis
Source: Biochem Genet. 2025 Jun 4;64(2):2488–507. doi: 10.1007/s10528-025-11151-x (PMC13086824; doi:10.1007/s10528-025-11151-x)
Supplement: Supplementary file 1 — Supplementary file1 (DOCX 2121 KB) [file 10528_2025_11151_MOESM1_ESM.docx]

**Supplementary material**

**Melt curve profiles:**

**
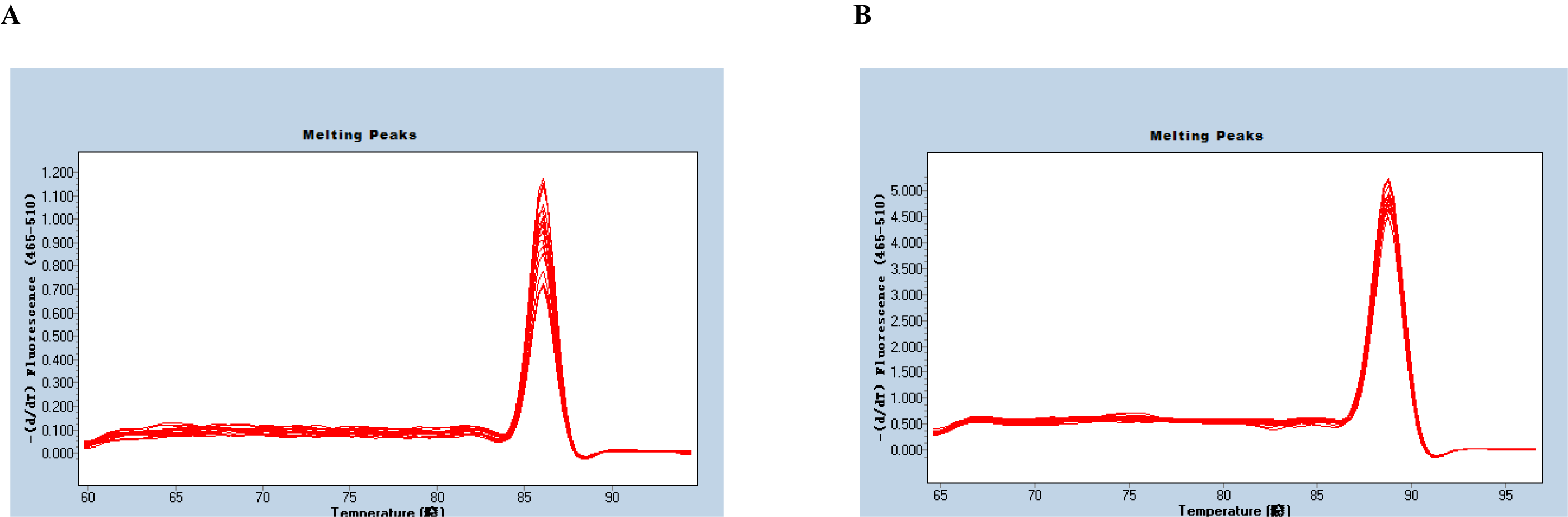
Figure.S1** Melt curves of GADPH(A) and CAV1(B).

**
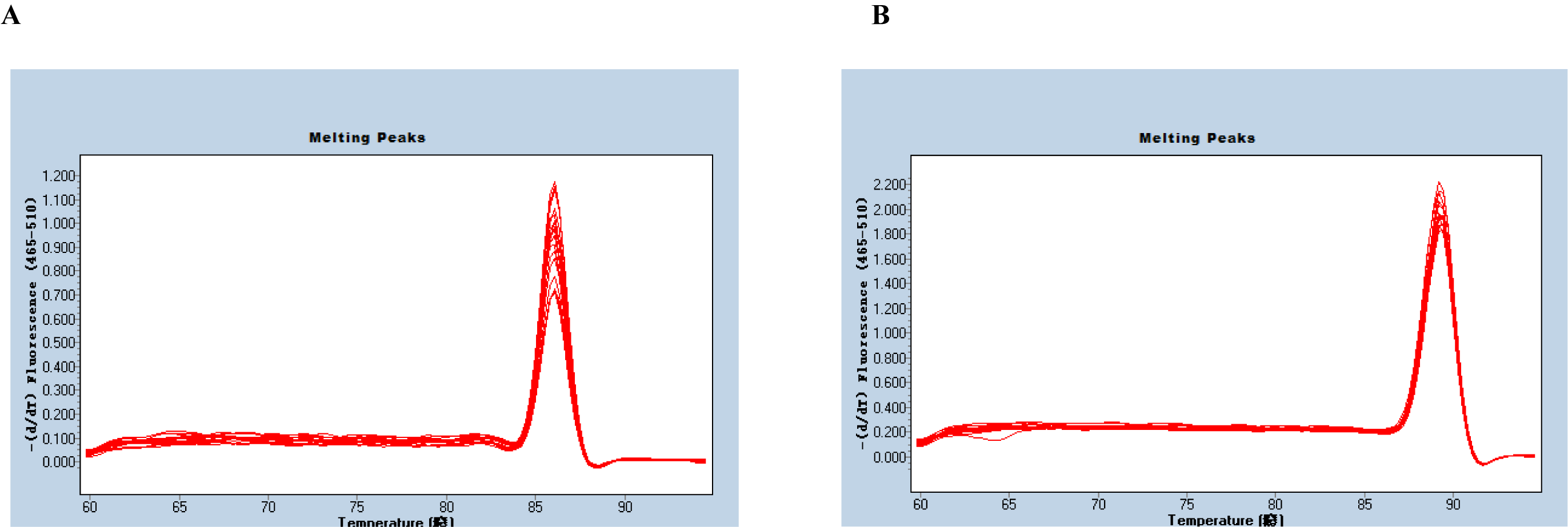
Figure.S2** Melt curves of GADPH(A) and PDK4(B).

**
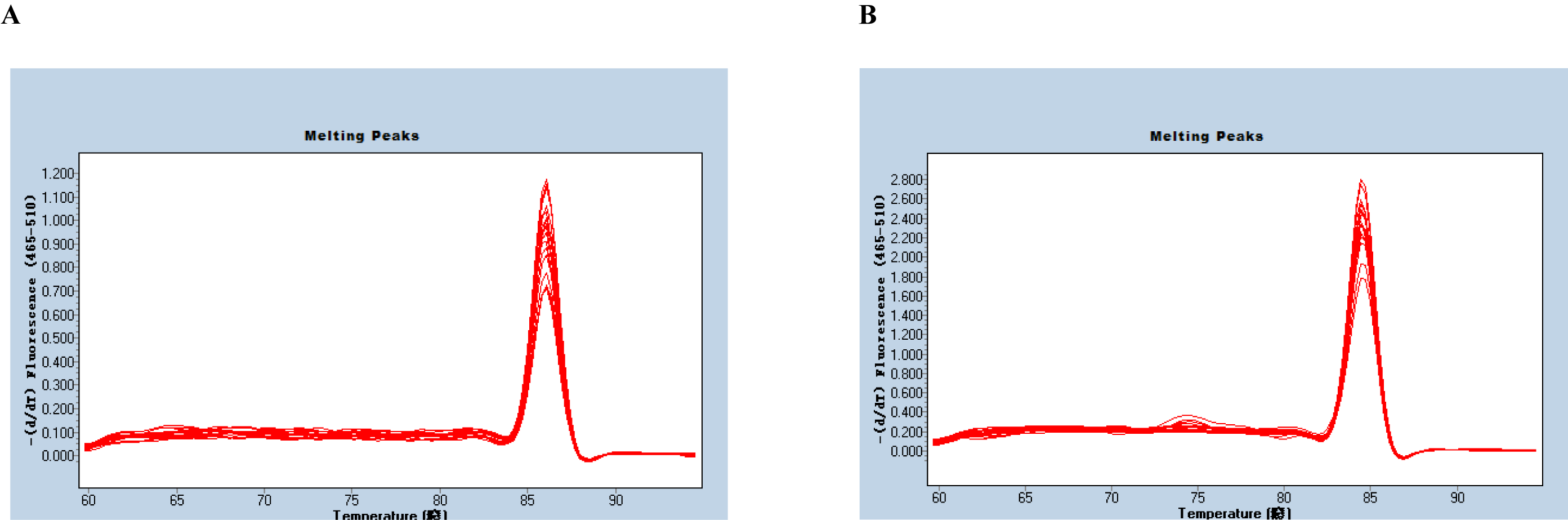
Figure.S3** Melt curves of GADPH(A) and CSGP4(B).


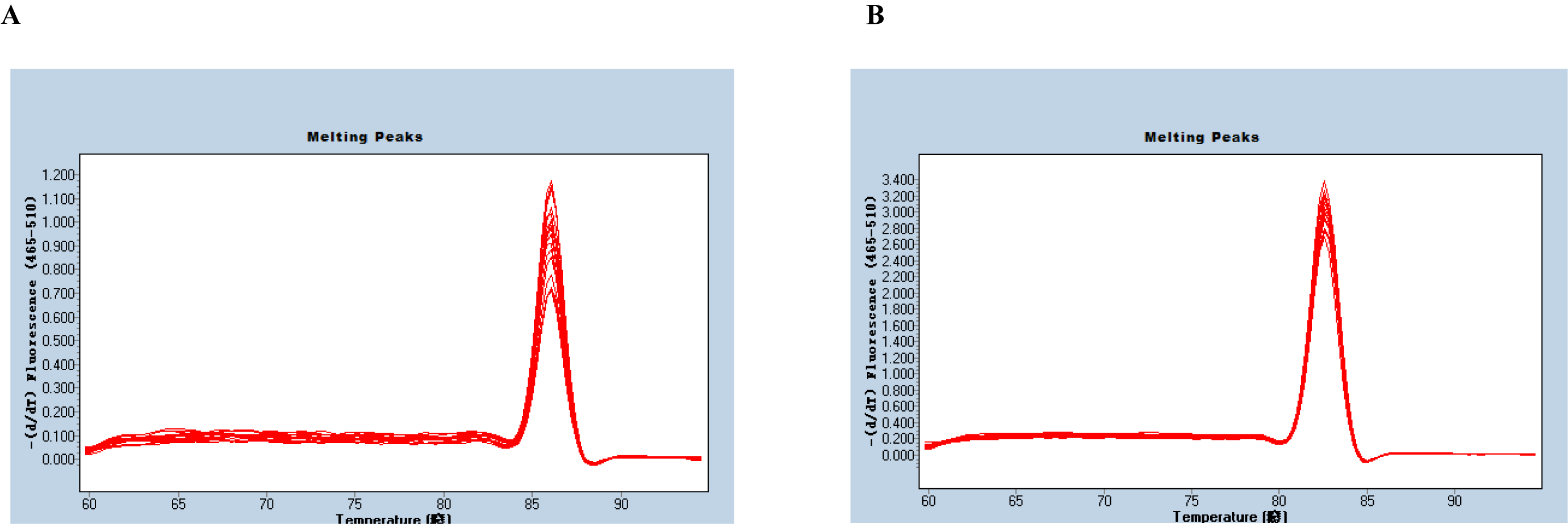
**Figure.S4** Melt curves of GADPH(A) and SERPINE1(B).
